# Improved Wound Healing of Cutaneous Sulfur Mustard Injuries in a Weanling Pig Model

**Published:** 2006-11-08

**Authors:** John S. Graham, Robert S. Stevenson, Larry W. Mitcheltree, Marcia Simon, Tracey A. Hamilton, Robin R. Deckert, Robyn B. Lee

**Affiliations:** a Medical Toxicology Branch, Analytical Toxicology Division, US Army Medical Research Institute of Chemical Defense, Aberdeen Proving Ground, MD; b Comparative Pathology Branch, Comparative Medicine Division, US Army Medical Research Institute of Chemical Defense, Aberdeen Proving Ground, MD; c Technical Support Branch, Administrative Division, US Army Medical Research Institute of Chemical Defense, Aberdeen Proving Ground, MD; d Department of Oral Biology & Pathology, State University of New York, Stony Brook, NY

## Abstract

**Objective:** The objective was to examine the efficacy of several treatment regimens in improving wound healing of cutaneous sulfur mustard (HD) injuries. **Methods:** Wound healing studies were conducted in weanling pigs. Superficial dermal HD injuries were debrided at 48 hours postexposure using an erbium-doped yttrium aluminum garnet (Er:YAG) laser, followed by application of a treatment adjunct. A variety of noninvasive bioengineering methods were conducted during the postsurgical observation period to examine the various cosmetic and functional aspects of the skin. Histopathology was performed at the end of each study (14 or 21 days postsurgery). **Results:** As noted clinically, reepithelialization was nearly complete by 7 days postsurgery for many of the sites treated with petrolatum and scarlet red dressings. By 21 days, the skin elasticity of the petrolatum-dressed sites was not significantly different from that of sham-exposed skin. Upon dressing removal on postsurgery day 4, the neoepidermis of allograft- and thin film-dressed sites was partially removed, with resultant petechial hemorrhaging. Mean pathology scores for hydrocolloid-dressed sites were significantly lower than those of untreated HD-exposed sites on postsurgery day 14. **Conclusions:** Care must be taken during bandage changes, and a nonadherent dressing that could be left in place for a longer period of time (eg, 7 days) would be beneficial. The use of cultured epithelial allograft material may have a potential role if grown on a completely nonadherent backing and left undisturbed for at least a week. Xeroform Petrolatum and Scarlet Red Ointment dressings are effective and inexpensive treatment adjuncts for HD injuries.

Significant cutaneous sulfur mustard (HD) injuries can take several months to heal, necessitate lengthy hospitalizations, and result in significant cosmetic and/or functional deficits. There are currently no standardized or optimized methods of casualty management. Current treatment strategy consists of symptomatic management and is designed to relieve symptoms, prevent infections, and promote healing.^1^–^5^ New strategies are needed to provide for optimal and speedy wound healing. The ultimate goal is to determine the most efficacious treatment regimen to be applied in the clinical management of HD casualties. The ideal regimen should return damaged skin to optimal appearance and normal function in the shortest time. Improved treatment will result in a better cosmetic and functional outcome for the patient and will enable the casualty to return to normal activities sooner.

As with deep thermal burns, deep HD injuries will require very aggressive approaches (eg, serial tangential excision followed by autologous split-thickness skin grafting).^6^,^7^ While past HD wound-healing research in our laboratory has concentrated on deep injuries, superficial dermal (second degree) HD injuries may have greater clinical relevance on the battlefield. Partial-thickness injuries will likely not require such surgically aggressive approaches. Treatment strategies for improved healing of established partial-thickness cutaneous HD injuries have recently been formulated.^8^,^9^ The approach we are taking is to perform adequate debridement of partial-thickness injuries, then treat the lesions like chronic cutaneous ulcers or partial-thickness thermal burns using contemporary medical approaches.^8^,^9^ Following adequate wound debridement using an erbium-doped yttrium aluminum garnet (Er:YAG) laser, one or more treatment adjuncts are applied. Examples of adjuncts under consideration are dressings, topical nutritional support, skin substitutes, keratinocyte sprays, and vacuum assisted closure (VAC).^8^,^9^

We describe here several treatment efficacy studies conducted in a weanling pig model. Similarities between human and porcine skin make this species an appropriate animal model for efficacy testing of candidate treatment regimens.^8^ In addition to the histopathology conducted at the end of the 14- to 21-day wound healing studies, a variety of noninvasive bioengineering methods were used throughout the healing period to assess treatment efficacy. Such methods included reflectance colorimetry to evaluate erythema, skin hue, chroma, and lightness^7^,^10^–^13^; torsional ballistometry to evaluate the mechanical properties of skin firmness and elasticity^7^; evaporimetry to examine transepidermal water loss as a way to evaluate barrier function^7^,^13^; 2-dimensional high-frequency (20 MHz) ultrasound to examine edema formation^11^ and scar tissue thickness; and image analysis to evaluate wound contraction.^6^

## MATERIALS AND METHODS

### Animal model

Female Yorkshire crossbred pigs (weanlings), *Sus scrofa*, 9 to 12 kg, were used (Country View Farms, Shanksville, Pa). Research was conducted in compliance with the Animal Welfare Act and other Federal statutes and regulations relating to animals and experiments involving animals and adhered to principles stated in the *Guide for the Care and Use of Laboratory Animals*, National Research Council, 1996. The facility where this research was conducted is fully accredited by the Association for Assessment and Accreditation of Laboratory Animal Care International.

### Sulfur mustard exposure

Eighteen to 24 hours before agent exposure, the ventral abdominal skin was clipped with an electric clipper, and then depilated as previously described.^7^ Four exposure sites were demarcated on the ventral abdominal surface, and exposed to liquid sulfur mustard for 8 min to produce superficial dermal injuries. The exposure procedures used were the same as those previously described to induce 3-cm-diameter HD injuries^7^; however, in place of the tile floats a calibration weight (300 g) was placed on top of the rubber stopper and manually held in place for the duration of the exposure to ensure even downward pressure and complete contact of the wetted filter paper with the skin.

### Debridement procedure

At 48 hours postexposure, the animals were anesthetized with isoflurane and selected lesions were debrided to a depth of 300 or 400 μm with an Er:YAG laser (Sciton Profile Laser Surgical System with Scanner, Sciton Inc, Palo Alto, Calif) then immediately covered with one of several dressings tested. On each animal, one HD-exposed site was left untreated (ie, no debridement), and the other 2 HD-exposed sites were debrided with the Er:YAG laser and a dressing applied. The fourth experimental (sham-exposed) site served as a negative control.

### Dressings

The following sterile dressings were tested: Xeroform Petrolatum Dressing (Sherwood Medical, St. Louis, Mo), Scarlet Red Ointment Dressing (Sherwood Medical, St. Louis, Mo), frozen cultured epithelial porcine allograft (Living Skin Bank, SUNY—Stony Brook, NY), Flexzan Foam Adhesive Dressing (Bertek Pharmaceuticals Inc, Morgantown, WVa), and DuoDERM Signal hydrocolloid dressing (ConvaTec, Princeton, NJ).

Xeroform Petrolatum Dressing and Scarlet Red Ointment Dressing were tested side by side in one wound healing study. Four pigs were used, with a 21-day healing period following surgery. Lesions were debrided to a depth of 400 μm in multiple passes of the laser. One laser-debrided site on each pig was dressed with Xeroform, and the other with Scarlet Red. These dressings were secured over the laser-debrided wounds using surgical staples. A pressure dressing was then applied using Kerlix Super Sponges (Tyco Healthcare Group LP, Mansfield, Mass), Reston Self-Adhering Foam Pads (3M HealthCare, St Paul, Minn), and elastic tape. The positive (+) control site (HD-exposed, not treated) and sham-exposed site (negative control, with no HD exposure) were not dressed. A 20-cm-wide tubular cotton stockinette (Abco Dealers Inc, Milwaukee, Wisc) was then stapled in place over the animal's entire torso to protect the dressings from becoming soiled. On the sixth day following surgery, these dressings were removed and replaced with Telfa Plus Barrier Island Dressings (Kendall Healthcare Products Company, Mansfield, Mass) and elastic tape, which were kept in place for one additional day. Sites remained undressed for the remainder of the experiment.

Frozen cultured epithelial porcine allograft and Flexzan Foam Adhesive Dressing were tested side-by-side in a second wound healing study. Six pigs were utilized, with a 14-day healing period following surgery. Lesions were debrided to a depth of 400 μm in multiple passes of the laser. One laser-debrided site on each pig was dressed with allograft, and the other with Flexzan. The cultured porcine allograft material was grown from pig keratinocytes collected from weanling pigs procured from the same vendor as the one that supplied pigs for these efficacy studies. Epidermis was separated from dermis using Dispase II (Roche Diagnostics GmbH, Mannheim, Germany).^14^ Keratinocytes were then harvested and serially cultured with lethally irradiated 3T3-J2 feeder cells as previously described^15^ with minor modifications.^16^ Allografts were then prepared on a petrolatum gauze backing and stored at −70°C according to the procedures of Tamariz *et al*.^17^ Keratinocyte isolation and allograft production was carried out at the State University of New York, Stony Brook. A frozen sheet of cultured porcine allograft was thawed to room temperature, placed over one laser-debrided HD-exposed wound, covered with a single layer of Xeroform gauze, and a pressure dressing put in place using Kerlix Super Sponges, Reston Self-Adhering Foam pads, and elastic tape. A single 4″ × 4″ sheet of Flexzan was placed over another laser-debrided HD-exposed site and covered with sterile gauze. The + control site and sham site were covered with sterile gauze, secured in place by surgical staples. A tubular stockinette was then stapled in place over the animal's entire torso to protect the dressings from becoming soiled. On the fourth day following surgery, all dressings were carefully removed and additional (new) dressings put in place over the sites dressed with allograft or Flexzan. On the sixth day following surgery, these dressings were removed and replaced with Telfa Plus Barrier Island Dressings (Kendall Healthcare Products Company, Mansfield, Mass) and elastic tape, which were kept in place for one additional day. Sites remained undressed for the remainder of the experiment.

DuoDERM Signal dressings were tested in a third wound healing study that involved 2 different depths of laser debridement (300 and 400 μm). Six pigs were utilized, with a 14-day healing period following surgery. One HD-exposed site on each animal was debrided with the Er:YAG laser to a depth of 400 μm in multiple passes. A second HD-exposed site had 300 μm of tissue removed in multiple passes of the laser. Both debrided sites (designated as DuoDERM-300 and DuoDERM-400) were dressed with DuoDERM Signal. While these dressings have an adhesive border, the edges of dressings were further secured with surgical tape (Durapore), stapled on the corners. The + control site and sham site were covered with sterile gauze (stapled in place with edges further secured with Durapore tape). A tubular stockinette was then stapled in place over the animal's entire torso to protect the dressings from becoming soiled. Dressings were removed on the seventh day following surgery. Sites remained undressed for the remainder of the experiment.

A summary flowchart for each experiment is presented below.

**Table:** 

EXPERIMENT*	Time of surgery (postexposure)	Debridement depth (μm)	2nd dressing application	Postsurgery (PS) day for pathology
Xeroform/Scarlet Red	48 h	400	NO	21
Allograft/Flexzan	48 h	400	Yes (PS04)	14
DuoDERM-300/400	48 h	300 or 400	No	14

*Each animal had 4 experimental sites: 1 sham-exposed site (−control), 1 HD-exposed and untreated site (+ control), and 2 HD-exposed and treated sites. Sites were rotated on the ventral abdominal surface to preclude sensitivity biases based upon anatomical location. Treated sites were laser-debrided to the specified depth, and the dressings applied. All dressings were removed 7 days after surgery. Noninvasive bioengineering measurements were conducted before HD exposure and weekly after surgery until euthanasia.

### Postsurgical procedures

#### Noninvasive bioengineering methods

A variety of noninvasive bioengineering methods were used to follow the progress of wound healing. Measurements included reflectance colorimetry (RC) to measure erythema, skin hue, chroma, and lightness; evaporimetry to examine transepidermal water loss (TEWL) as a method of evaluating barrier function; torsional ballistometry (TB) to evaluate the mechanical properties of skin firmness and elasticity; and 2-dimensional high-frequency (20 MHz) ultrasonography (HFU) to monitor skin thickness. Measurements were made before agent exposure and on postsurgery days 7 (PS07; all studies), 14 (PS14; all studies), and 21 (PS21; Xeroform/Scarlet Red study only) for all methods except HFU, which was only conducted before exposure and on the last day of each study (PS14 or PS21). Torsional ballistometry was not conducted on PS07 in the DuoDERM study, to avoid disruption of the fragile neoepidermis. HFU was not conducted in the DuoDERM study because of equipment failure. Bioengineering methods were performed as previously described.^7^

#### Digital photography

Digital pictures of gross lesions were taken using a Kodak Professional DCS 315 digital camera (Eastman Kodak Company, Rochester, NY). Images were used to assess wound contraction by image analysis on PS07 (all studies), PS14 (all studies), and PS21 (Xeroform/Scarlet Red study only).

#### Pathology

On the last day of each study, animals were euthanized with an overdose of pentobarbital sodium (Fatal-Plus Solution, Vortech Pharmaceuticals Ltd, Dearborn, Mich; 78 mg/kg i.v.). Full-thickness excisions (including panniculus carnosa) of each entire lesion plus surrounding skin were removed, stapled onto labeled, laminated index cards, and placed into 10% neutral buffered formalin. Sections were later trimmed, paraffin embedded, cut on a microtome into 5-μm-thick sections, and stained with hematoxylin and eosin (H&E) for routine histopathology. Serial sections were also stained with Masson's trichrome to highlight dermal collagen, and Movat's pentachrome to highlight elastic fibers. A veterinary pathologist scored the sections in a blinded fashion based on a published histomorphologic scale for rating burn scars^18^ modified for evaluation of the tissues. The following scoring system was used (highest number represented normalcy): reepithelialization (1 = complete, 0 = not complete), epidermal hyperplasia (1 = absent, 0 = present), epidermal-dermal separation (1 = absent, 0 = present), inflammatory cells (1 = absent, 0 = present), hair follicles (1 = present, 0 = absent), glands (1 = present, 0 = absent), elastic fibers (1 = normal, 0 = reduced size and number), smooth muscles (1 = present, 0 = absent), collagen orientation (3 = normal, 2 = abnormal papillary, 1 = abnormal upper reticular, 0 = abnormal lower reticular), fibroplasia (1 = absent, 0 = present), vascular proliferation (1 = absent, 0 = present), and hemorrhage (1 = absent, 0 = present). Maximum total score for best possible outcome (eg, normalcy) was 14.

### Statistics

#### Xeroform/Scarlet Red study

For the bioengineering data, comparison of the experimental groups (sham controls, + controls, and treated groups) and days of observation were made for each measurement using a 3-factor mixed-model analysis of variance (ANOVA) using SPSS 13.0 for Windows (SPSS Inc, Chicago, Ill). The 2 fixed factors were experimental groups and days of observation. The random factor was pig number. If a significant experimental group by day interaction was observed, a 2-factor mixed-model ANOVA was used at each day to compare experimental groups. The fixed factor was experimental groups and the random factor was pig number. Pathology data were compared between experimental groups using a Chi-square test on the frequencies of presence/absence and other categorical scores, followed by either a Chi-square or Fisher exact test to compare pairs of experimental groups. A one-factor ANOVA followed by a Tukey test was used to compare the mean total score parameter between experimental groups. Statistical significance was defined as *p* < .05 for all tests.

#### Allograft/Flexzan and DuoDERM studies

For the bioengineering data, sites were compared statistically using a one-factor ANOVA at each day within each experimental group to compare responses between sites. A 2-factor repeated-measures ANOVA with repeated measurements on both factors, day and experimental group, was used to compare the measurements made on the exposure sites for each experimental group. If a significant treatment by day interaction was observed, then a randomized block ANOVA at each day was used to compare the experimental groups. Pathology data was compared between experimental groups using a Chi-square test on the frequencies of presence/absence, followed by either a Chi-square or Fisher exact test to compare pairs of experimental groups. ANOVA and *t* tests were used for the total score parameter.

## RESULTS

### Xeroform/Scarlet Red study

Mean animal weight on the day of exposure was 8.8 kg. Mean weight at the end of the study (PS21) was 10.8 kg.

#### Transepidermal water loss

**Figure 1:**
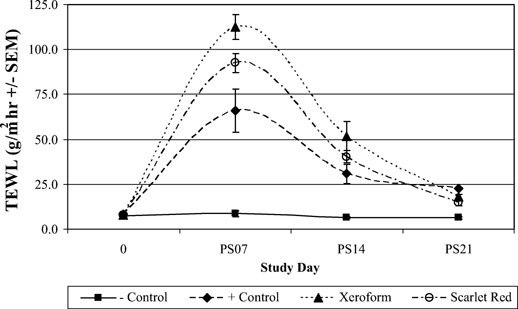
Transepidermal water loss (TEWL) rates from the Xeroform/Scarlet Red study. Barrier function was significantly disrupted for all HD-exposed groups on PS07 and PS14. By PS21, barrier function of all HD-exposed groups had improved and was approaching normal limits of transepidermal water loss. At PS21, the + control group had significantly higher water loss rates than did the sites dressed with Xeroform or Scarlet Red.

No significant treatment group differences were noted before exposure (Fig 1). Bioengineering data indicated barrier function was significantly disrupted for all HD-exposed groups at PS07 and PS14. At PS07 and PS14, the mean TEWL rate for the sham group was significantly less than for all other treatment groups, and the mean TEWL rate for the + control group was significantly less than for the Xeroform group. Barrier function of all HD-exposed groups had improved and was approaching normal limits of transepidermal water loss by PS21. For the sham group, the mean TEWL rate was significantly lower at PS21 than for all other groups, and the + control group had significantly higher water loss rates than did the sites treated with Xeroform or Scarlet Red.

#### Reflectance colorimetry

Reflectance colorimetry could not be adequately evaluated because the red ointment from the Scarlet Red dressing stained the skin. Data are not shown.

#### Torsional ballistometry

**Figure 2:**
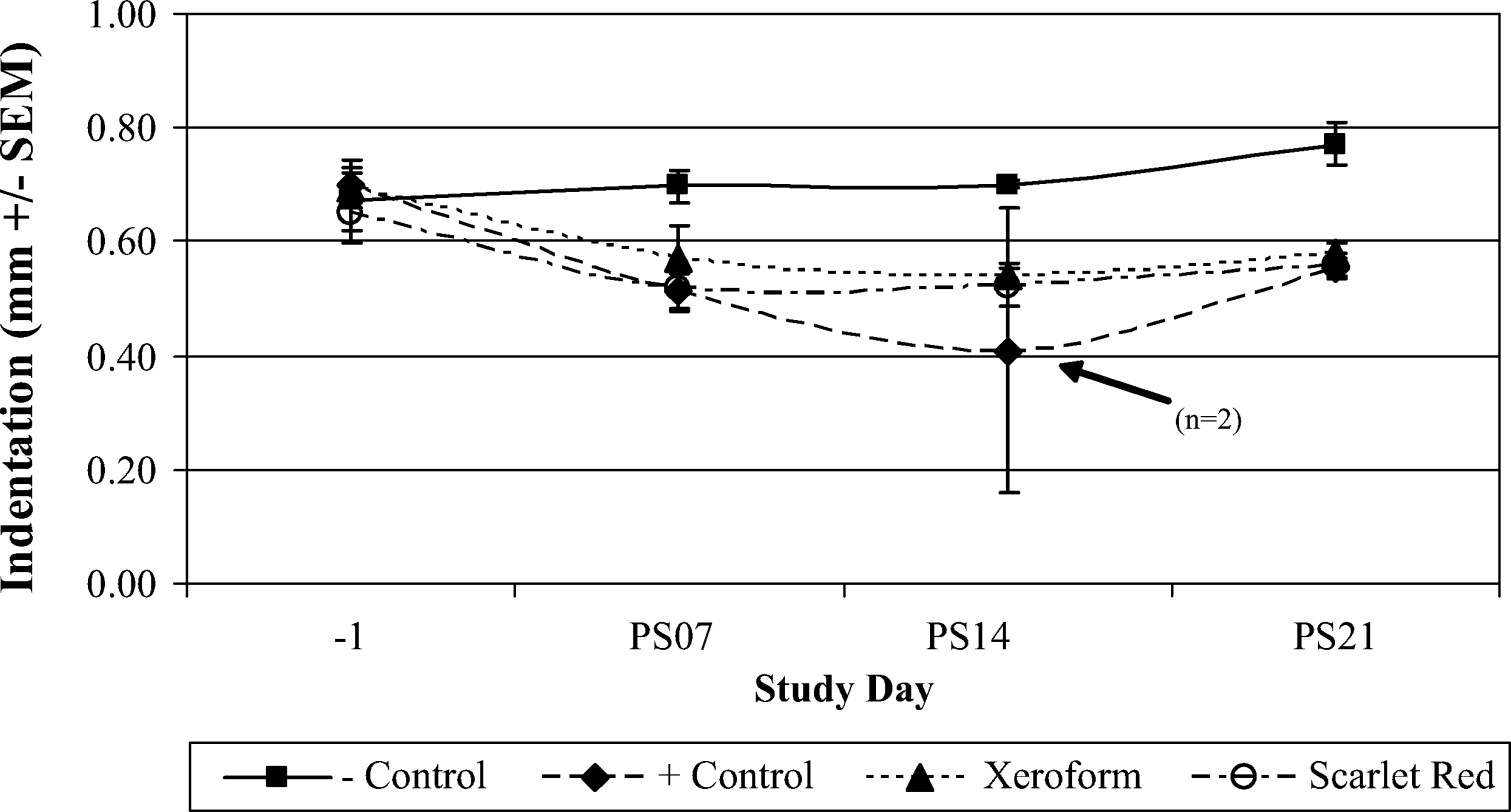
Torsional ballistometry measurements for skin hardness from the Xeroform/Scarlet Red study. On PS07 and PS21, indentation of the sham-exposed (– control) group was significantly greater than the other groups, indicating softer skin.

**Figure 3:**
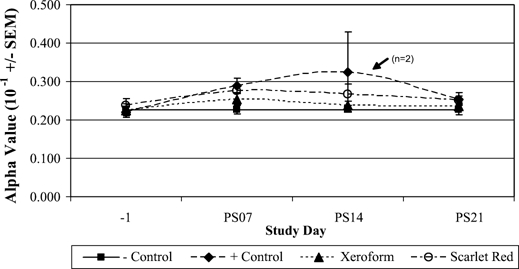
Torsional ballistometry measurements for skin elasticity from the Xeroform/Scarlet Red study. On PS21, the α values for the untreated positive control and Scarlet Red groups were significantly greater than for the sham group, indicating less elasticity. Skin elasticity of the Xeroform-treated sites was not significantly different from that of the sham-exposed skin (– controls).

For the torsional ballistometry parameters, the + control group had 2 outliers on PS14, which affected data analysis; therefore, PS14 data were excluded. For mean indentation values (Fig 2), a significant day by treatment group interaction was noted. No significant differences were noted before exposure. On PS07 and PS21, indentation of the sham group was significantly greater than the other groups, indicating softer skin. No significant differences in mean α measurements (Fig 3) were noted before exposure or on PS07. On PS21, the mean α values for + control and the Scarlet Red groups were significantly greater than for the sham group, indicating less elasticity. Skin elasticity of the Xeroform-treated sites was not significantly different from that of sham-exposed skin.

#### High-frequency ultrasonography

**Figure 4:**
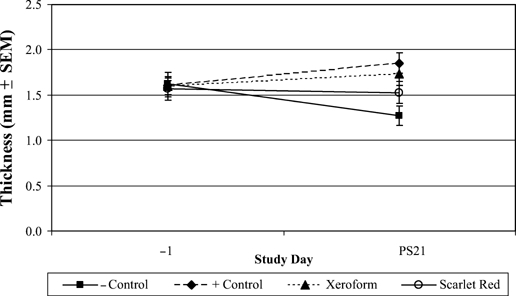
High frequency ultrasound measurements for skin thickness from the Xeroform/Scarlet Red study. At PS21, skin from the sham-exposed (– control) group was significantly less thick than the skin from all other groups. Mean thickness of the Scarlet Red group was significantly less than the + control and Xeroform groups.

No significant differences were observed in skin thickness between treatment groups before exposure (Fig 4). At PS21, the sham-exposed group was significantly less thick than the + control, Xeroform and Scarlet Red groups. Mean thickness of the Scarlet Red group was significantly less than the + control and Xeroform groups.

#### Wound contraction

No apparent wound contraction was noted after wound debridement in any experimental group (data not shown).

#### Pathology

**Figure 5:**
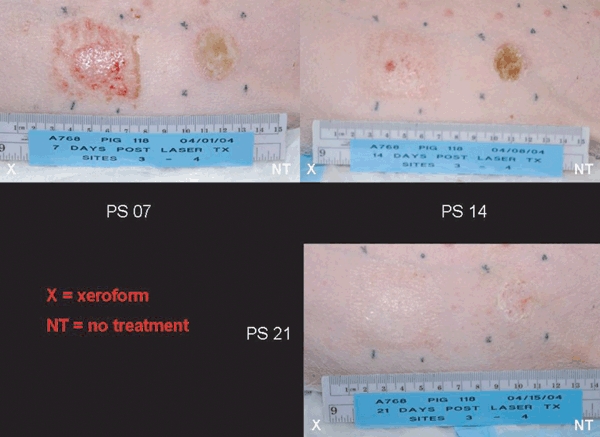

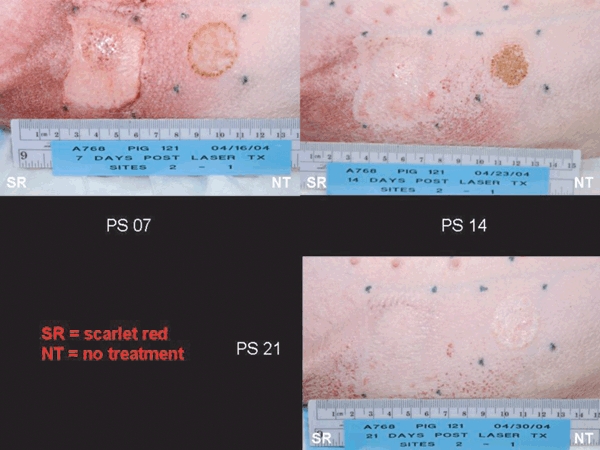
Clinical pictures on PS07, PS14, and PS21from the Xeroform/Scarlet Red study. (a) The Xeroform sites had grossly reepithelialized between PS07and PS14, whereas the untreated (NT) HD-exposed sites had a thin dry eschar present on PS14 that was just beginning to separate from the underlying, regenerating epithelium. (b) The Scarlet Red sites had grossly reepithelialized between PS07 and PS14, whereas the untreated (NT) HD-exposed sites had a thin dry eschar present on PS14 that was just beginning to separate from the underlying, regenerating epithelium. Small spider veins were noted along portions of the periphery of several of the Scarlet Red–dressed sites.

**Figure 6:**
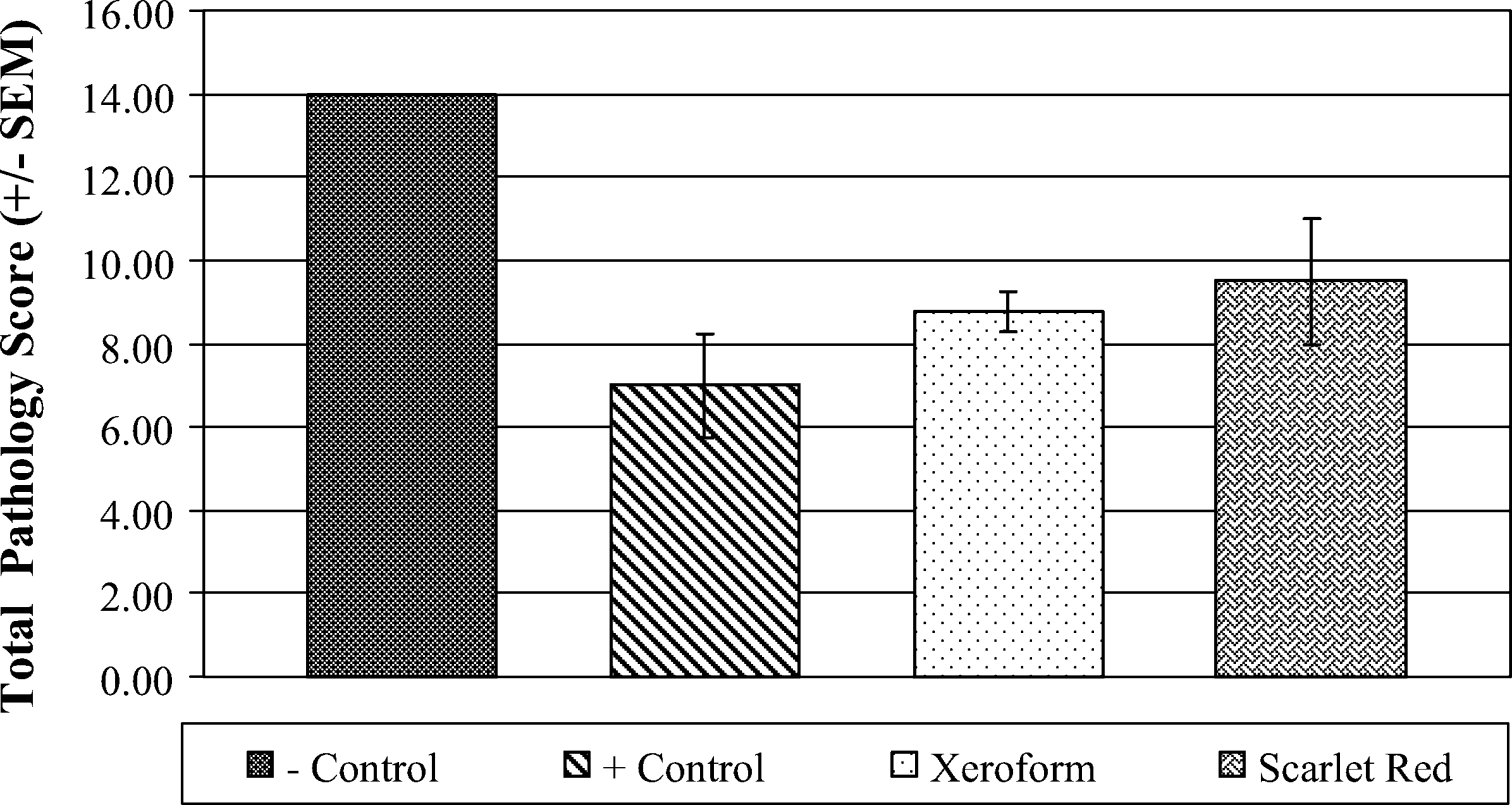
Total pathology scores from the Xeroform/Scarlet Red study. The Xeroform- and Scarlet Red–dressed sites had higher mean total scores than did the + control (untreated) sites; however, statistical significance was not achieved.

Both the Xeroform- and Scarlet Red–treated sites had grossly reepithelialized between PS07 and PS14, whereas the untreated HD-exposed sites had a thin dry eschar present on PS14 which was just beginning to separate from the underlying, regenerating epithelium (Figs 5a and 5b). No signs of infection were noted. Small spider veins were noted along portions of the periphery of several of the debrided sites that were dressed with Scarlet Red (Fig 5b). Histopathology indicated that for epidermal hyperplasia, elastic fibers, collagen orientation, fibroplasias, vascular proliferation, and hemorrhage, the sham group had significantly different distributions of responses from the + control group (data not shown). For hyperplasia, elastic fibers, and fibroplasias, the sham group had significantly different distributions of responses from the Xeroform group. No other significant differences were observed. For total score (Fig 6), the + control, Scarlet Red, and Xeroform groups had significantly lower mean scores than the sham group. The Xeroform- and Scarlet Red–dressed sites had higher mean total scores than the + control sites (high score of 14 = normalcy); however, statistical significance was not achieved. No other significant differences were observed.

### Allograft/Flexzan study

Mean animal weight on the day of exposure was 10.5 kg. Mean weight at the end of the study (PS14) was 14.9 kg.

#### Transepidermal water loss

**Figure 7:**
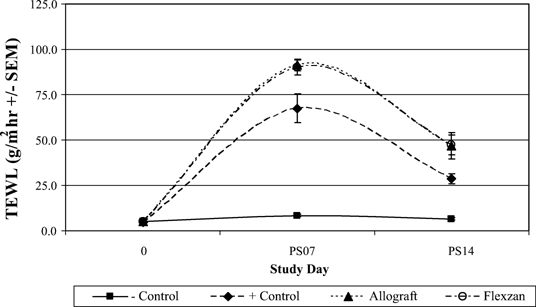
Transepidermal water loss (TEWL) rates from the allograft/Flexzan study. For PS07 and PS14, the sham-exposed (– control) sites had significantly lower TEWL rates than the + control, allograft, and Flexzan sites. Also, for PS07 and PS14, the + control sites had significantly lower TEWL rates than the allograft and Flexzan sites. TEWL rates for all HD-exposed groups decreased between PS07 and PS14.

No significant differences in transepidermal water loss rates were noted before exposurej (Fig 7). For PS07 and PS14, the sham sites had significantly lower TEWL rates than the + control, allograft, and Flexzan sites. Also, for PS07 and PS14, the + control sites had significantly lower TEWL rates than the allograft and Flexzan sites. TEWL rates for all HD-exposed groups decreased between PS07 and PS14.

#### Reflectance colorimetry

Significant differences among treatment groups were observed for L* on PS07, a* on PS07 and PS14, delta a* on PS07 and PS14, b* on PS07 and PS14, delta b* on PS07, C* on PS07 and PS14, and delta C* on PS07 (data not shown). In general, by PS14 the HD-exposed sites (treated and untreated) were more erythematous than the sham sites and were not significantly different in overall color change (delta E*_ab_) from each other.

#### Torsional ballistometry

**Figure 8:**
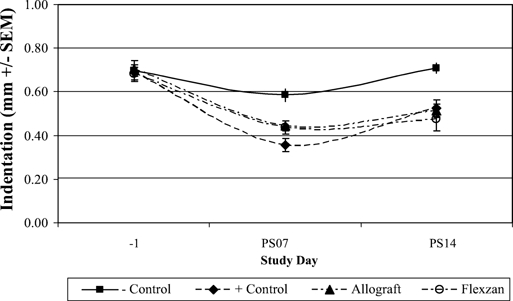
Torsional ballistometry measurements for skin hardness from the allograft/Flexzan study. By PS14, sites from the sham-exposed (– control) group were significantly softer than those in each of the other experimental groups.

**Figure 9:**
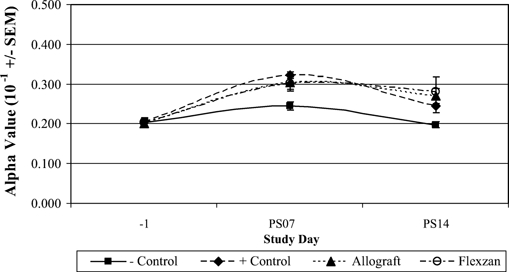
Torsional ballistometry measurements for skin elasticity from the allograft/Flexzan study. There were no differences in elasticity noted on PS14.

No significant differences in hardness or elasticity were noted before exposure (Figs 8 and 9). On PS07, the sham-exposed control was significantly softer than each of the other experimental groups, and the + control group was significantly harder than the allograft and Flexzan groups. The + control group was significantly less elastic than the sham group. On PS14, sites from the sham-exposed group were significantly softer than those in each of the other experimental groups. There were no differences in elasticity noted on PS14.

#### High-frequency ultrasonography

**Figure 10:**
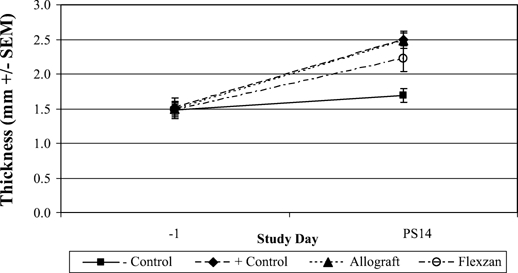
High frequency ultrasound measurements for skin thickness from the allograft/Flexzan study. On PS14, the sham-exposed (– control) sites were significantly less thick than the + control, allograft, and Flexzan sites.

No significant differences in skin thickness were noted before exposure (Fig 10). On PS14, the sham sites were significantly less thick than the + control, allograft, and Flexzan sites.

#### Wound contraction

No apparent wound contraction was noted after wound debridement in any experimental group (data not shown).

#### Pathology

**Figure 11:**
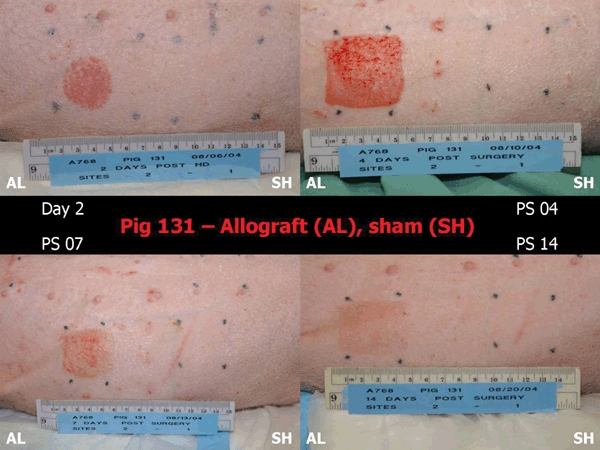

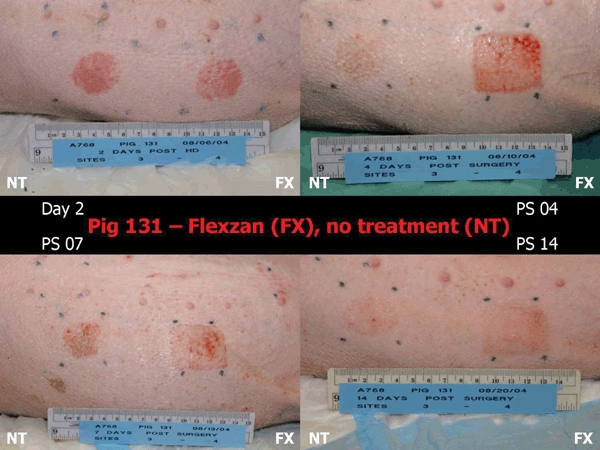
Clinical pictures on PS04, PS07, and PS14 from the allograft/Flexzan study. (a) Upon dressing removal on PS04, the neoepidermis of the allograft-dressed sites was partially removed, with resultant petechial hemorrhaging. (b) Upon dressing removal on PS04, the neoepidermis of the Flexzan-dressed sites was partially removed, with resultant petechial hemorrhaging.

**Figure 12:**
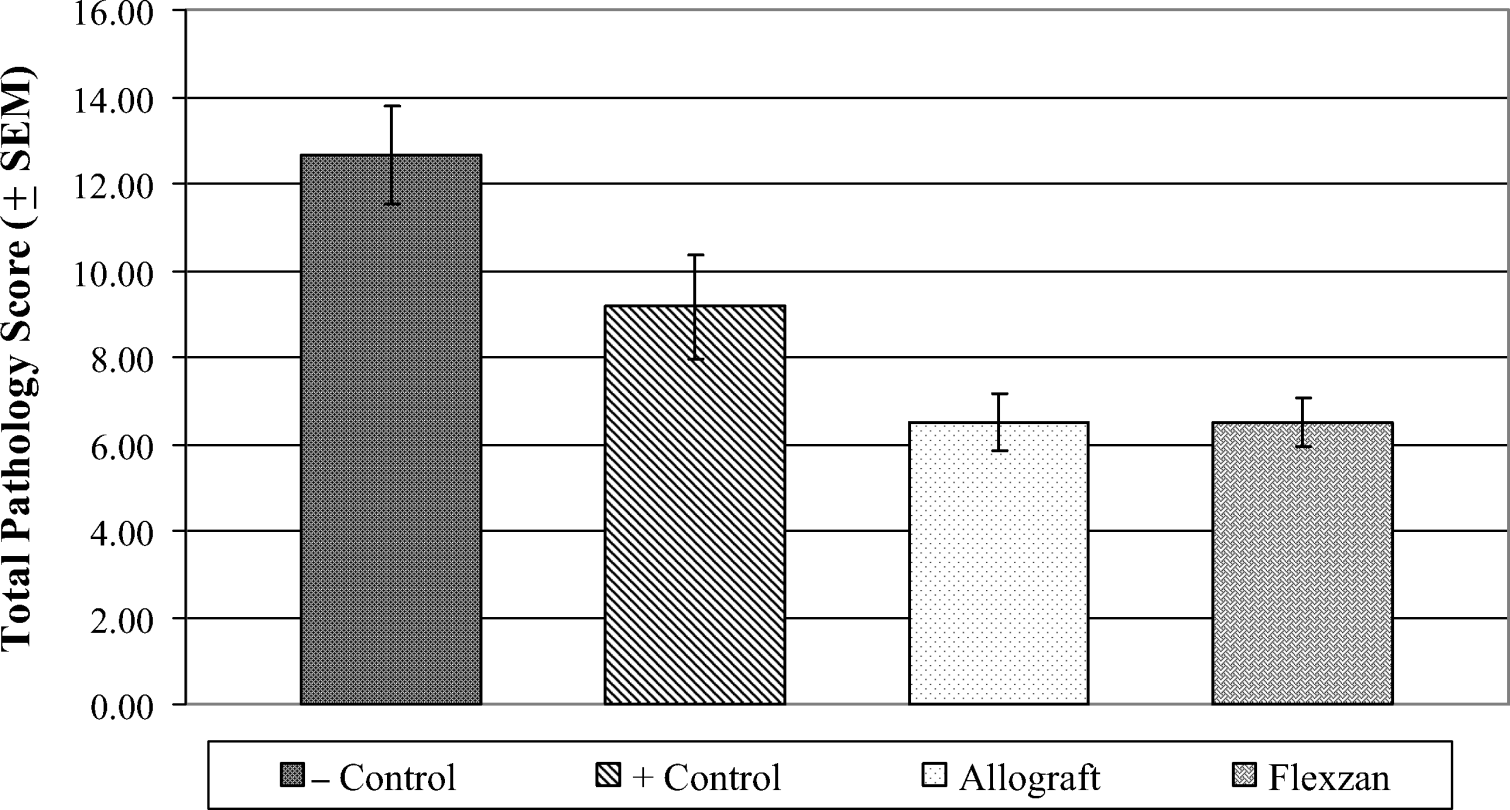
Total pathology scores from the allograft/Flexzan study. The allograft and Flexzan sites had significantly lower mean scores than the sham-exposed (– control) sites. No significant differences were observed between the + controls and the shams, allograft sites, and Flexzan sites.

Typical gross lesions can be seen in Figs 11a and 11b. Upon dressing removal on PS04, the neoepidermis of the allograft and Flexzan sites was partially removed, with resultant petechial hemorrhaging. No signs of infection were noted. The sham-exposed sites had significantly different distributions of responses for hyperplasia, collagen orientation, and vascular proliferation than did the allograft and Flexzan sites (data not shown). The sham sites also had a significantly different distribution of responses for fibroplasias than did the Flexzan sites. No other significant differences were observed. For total score (Fig 12), the allograft and Flexzan sites had significantly lower mean scores than the sham sites. No significant differences were observed between the + controls and the shams, allograft sites, and Flexzan sites.

### DuoDERM Signal study

Mean animal weight on the day of exposure was 12.3 kg. Mean weight at the end of the study (PS14) was 17.8 kg.

#### Transepidermal water loss

**Figure 13:**
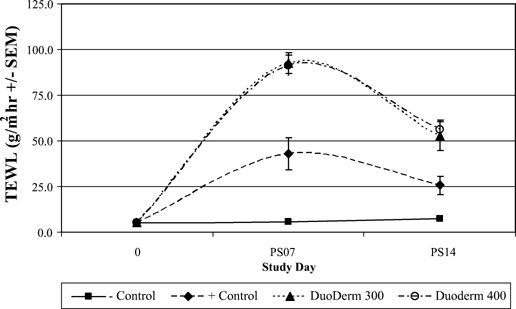
Transepidermal water loss (TEWL) rates from the DuoDERM study. For PS07 and PS14, the sham-exposed (– control) sites had significantly lower TEWL rates than the other sites. At these time points, the + control sites had significantly lower TEWL rates than the DuoDERM-300 and DuoDERM-400 sites. TEWL rates in all HD-exposed groups decreased between PS07 and PS14, indicating that barrier repair processes were underway.

No significant differences in transepidermal water loss rates were noted before exposure (Fig 13). For PS07 and PS14, the sham sites had significantly lower TEWL rates than the + control, DuoDERM-300, and DuoDERM-400 sites. Also, for PS07 and PS14, the + control sites had significantly lower TEWL rates than the DuoDERM-300 and DuoDERM-400 sites. TEWL rates in all HD-exposed groups decreased between PS07 and PS14, indicating that barrier repair processes were underway.

#### Reflectance colorimetry

Significant differences among treatment groups were observed for L* on PS07 and PS14, a* on PS07 and PS14, delta a* on PS07, b* on PS07, delta b* on PS07, and C* on PS07 and PS14 (data not shown). In general, by PS14 the + control sites were darker than the others, and all HD-exposed sites (treated and untreated) were more erythematous than were the sham sites. The HD-exposed sites (treated and untreated) were not significantly different in overall color change (delta E*_ab_) from each other.

#### Torsional ballistometry

**Figure 14:**
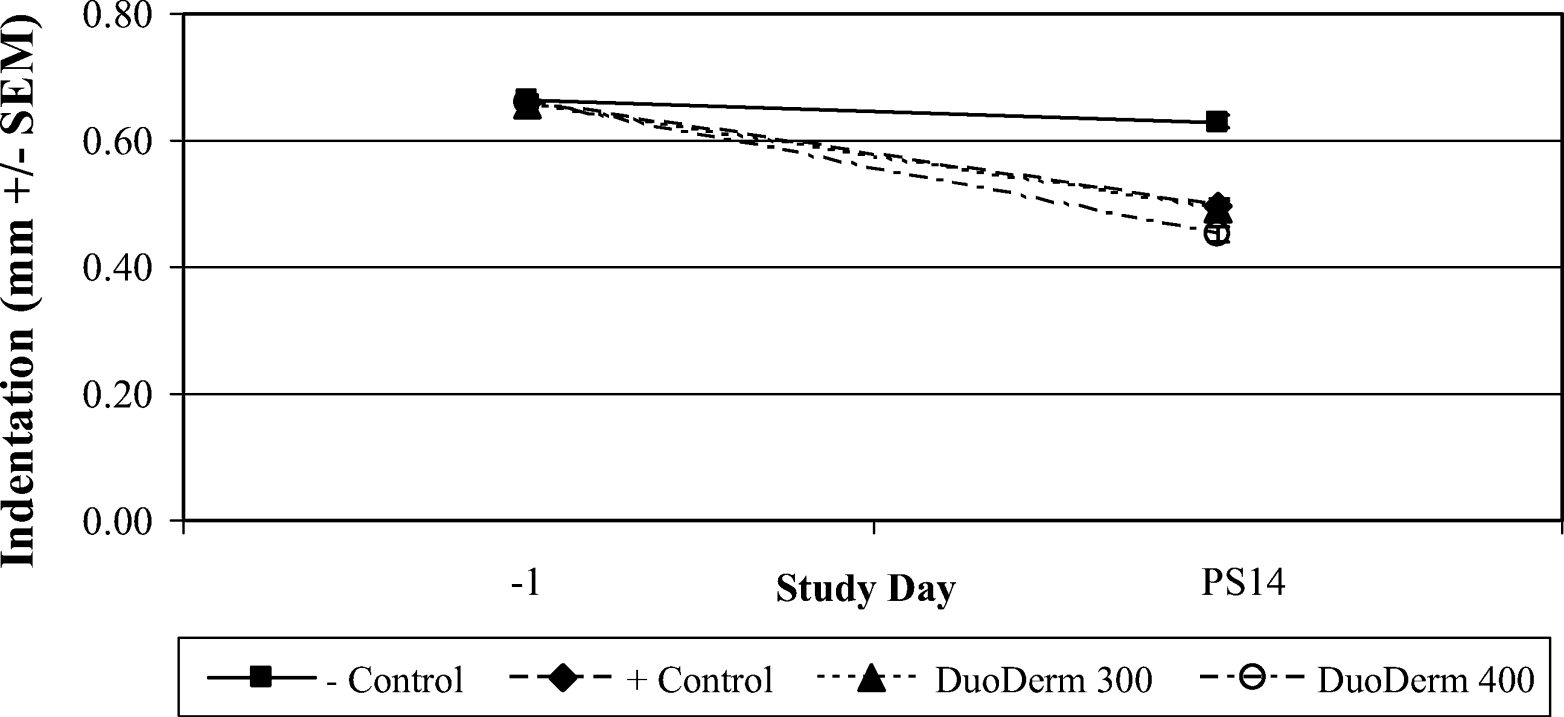
Torsional ballistometry measurements for skin hardness from the DuoDERM study. On PS14 the sham-exposed (– control) sites were significantly softer than those from the other experimental groups.

**Figure 15:**
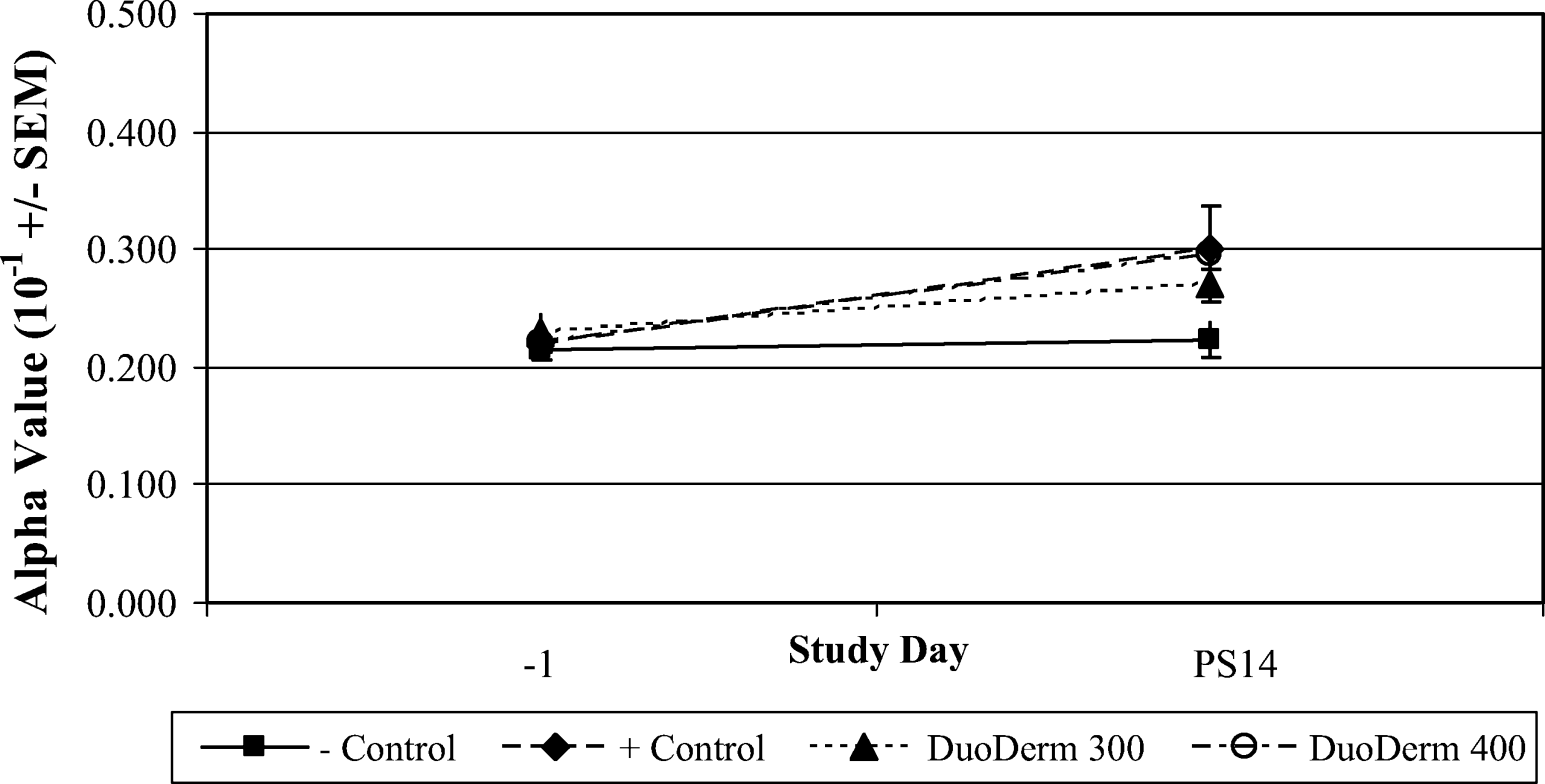
Torsional ballistometry measurements for skin elasticity from the DuoDERM study. On PS14, sites from the sham-exposed (– control) group were more elastic than were sites from the + control and DuoDERM-400 groups.

No significant differences in hardness or elasticity were noted before exposure (Figs 14 and 15). Because of the frailty to the neoepidermis after dressings are removed, these measurements were not conducted on PS07. On PS14, the sham sites were significantly softer than those from the other experimental groups. Sites from the sham-exposed group were more elastic than were sites from the + control and DuoDERM-400 groups.

#### High-frequency ultrasonography

Ultrasound measurements for skin thickness were not conducted because of equipment failure.

#### Wound contraction

No apparent wound contraction was noted after wound debridement in any experimental group (data not shown).

#### Pathology

**Figure 16:**
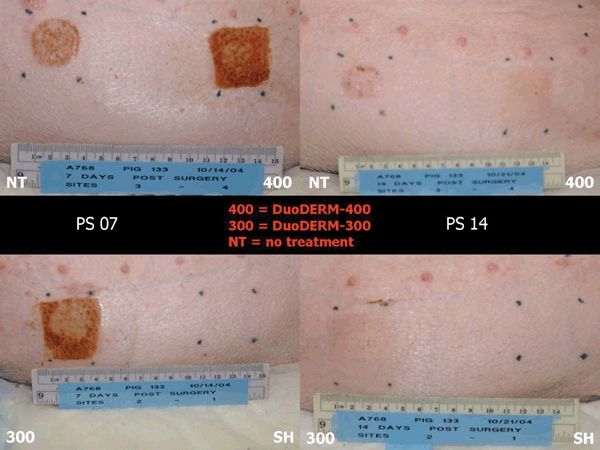
Clinical pictures on PS07 and PS14 from the DuoDERM study. Adhered elastic material (possibly hydrocolloid from the dressing) can be seen over the DuoDERM-treated sites on PS07.

**Figure 17:**
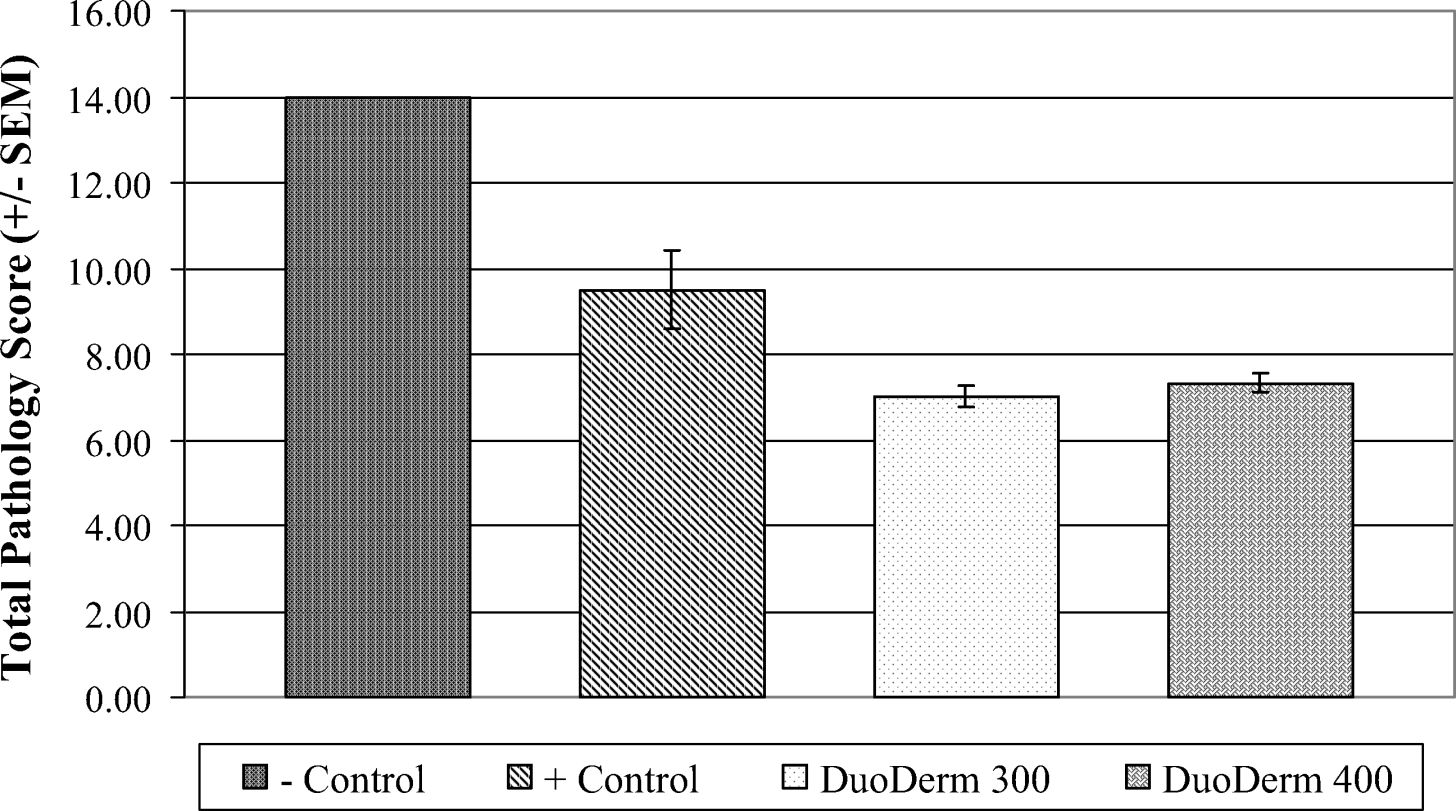
Total pathology scores from the DuoDERM study. The + controls, DuoDERM-300, and DuoDERM-400 sites had significantly lower mean scores than the sham-exposed (– control) sites, and the + controls had a significantly higher mean score than the DuoDERM-300 and DuoDERM-400 sites.

Typical gross lesions can be seen in Fig 16. Adhered elastic material (possibly hydrocolloid from the dressing) can be seen over the DuoDERM-300 and DuoDERM-400 sites. No signs of infection were noted. The sham sites had a significantly different distribution of responses for hyperplasia and inflammatory cells than did the + controls, DuoDERM-300 sites, and DuoDERM-400 sites (data not shown). The sham sites had a significantly different distribution of responses for elastic fibers, collagen orientation, fibroplasias, and vascular proliferation than did the DuoDERM-300 and DuoDERM-400 sites. The sham sites had a significantly different distribution of response for hemorrhage than did the + control sites. The + control sites had a significantly different distribution of responses for elastic fibers than did the DuoDERM-400 sites, and a significantly different distribution of responses for collagen orientation and vascular proliferation than did the DuoDERM-300 and DuoDERM-400 sites. For total scores (Fig 17), the + controls, DuoDERM-300 sites, and DuoDERM-400 sites had significantly lower mean scores than the sham sites, and the + controls had a significantly higher mean score than the DuoDERM-300 and DuoDERM-400 sites.

## DISCUSSION

Several sterile dressings were tested for efficacy in improving wound healing of cutaneous HD injuries following laser debridement. Xeroform is a petrolatum-based dressing that is commonly used in thermal burns management. Scarlet Red is a dressing that is sometimes used to dress split-thickness skin graft donor sites, providing speedy reepithelialization of donor sites for subsequent harvesting of additional graft material. Flexzan is a sterile, ultrathin, highly comfortable, semiocclusive polyurethane foam adhesive dressing that protects wounds from contamination and trauma while maintaining a moist wound healing environment. It is constructed of an open-cell foam with a closed-cell outer surface. Excess wound moisture is absorbed into the cells of the foam and allowed to evaporate through the outer surface, helping prevent fluid accumulation under the dressing. This dressing is frequently used following laser facial resurfacing. DuoDERM Signal is a tapered-edge hydrocolloid dressing that provides a moist wound healing environment and absorbs wound exudate. The dressing has a change indicator that “signals” the healthcare provider that enough wound exudate has been absorbed to require a dressing change. Cultured epithelial allografts and autografts have been used for about 2 decades as a treatment for chronic ulcers and thermal burns.^19^–^25^ Keratinocytes can be harvested from skin biopsies and grown to confluence by the method originally described by Rheinwald and Green.^15^ Large amounts of stratifying epidermis can thus be grown in the laboratory in short periods of time and used to restore defects in the epidermis.^26^ Such grafts can be used immediately or cryopreserved and used at a later date. In addition to their usefulness in improving the healing of deep ulcers and burns, they have shown efficacy in improving the rate of reepithelialization of partial-thickness burns^20^,^22^,^24^ and split-thickness skin graft donor sites.^21^,^23^,^25^ There is no evidence that cultured allografts survive permanently on the wound bed.^21^ Kaawach et al^20^ showed that allografted cells were not present between 8 and 100 days postgrafting and suggested that the newly formed epithelium was of host origin. Cultured keratinocyte allografts speed healing by providing cover and producing growth factors and extracellular matrix proteins.^23^ Because these coverings can be produced in large quantities and would thus be more readily available than cadaver skin, their application in the treatment of debrided partial-thickness HD injuries should be considered. Cultured epidermal autografts (CEA) would be safer to use, from the perspective of disease transmission, and would not require donor screening procedures. They do, however, require small punch biopsies to be collected from the patient and a lag time of about 2 weeks to grow the graft material. Despite their theoretical usefulness, CEA are rather limited in their clinical effectiveness because they are unable to withstand even very low levels of bacterial wound contamination and do not provide a durable epithelial surface. Wounds covered by this modality are unstable and are subject to frequent epithelial disruption as a result of minor mechanical trauma. Durability has been increased by placing the CEA on a scaffolding of widely meshed autograft.^27^ Alternatively, CEA placed over deepithelialized allograft (ie, engrafted allodermis) has also proved successful.^28^

In general, all HD-exposed sites (treated and untreated) had reepithelialized by PS14. Reepithelialization was nearly complete by PS07 for many of the sites treated with Xeroform and Scarlet Red. The pressure dressing applied over these 2 dressings may have played a role in these improved results by keeping the dressings in very firm contact with the prepared (laser-debrided) beds and reducing edema. In comparison, on PS14 the untreated HD lesions often had a hardened eschar that was just beginning to separate from the underlying, regenerating epithelium. Small spider veins were noted along portions of the periphery of several of the debrided sites that were dressed with Scarlet Red. While these small surface veins are not cosmetically desirable, they could be removed with further laser treatments (eg, via pulsed dye lasers^29^). It was noted that upon bandage changes on PS04 in the allograft/Flexzan experiment, some of the neoepidermis had adhered to the dressings and was removed with the dressings. Within the first week of surgery, the neoepidermis appears to be very fragile and easily removed. Care must be taken during bandage changes, and a nonadherent dressing that could be left in place for a longer period of time (eg, 7 days) would be beneficial, both to the patient and medical logistical burden (eg, nursing care). Several Flexzan-dressed sites were also noted to have a small accumulation of exudate built up underneath the dressing. This dressing was thus not able to adequately manage the exudate. The DuoDERM study showed that the amount of exudate produced by these laser-debrided lesions was greater than anticipated, as witnessed by the amount of fluid absorbed into the hydrocolloid dressing. The amount of exudate was moderate and the DuoDERM dressing was able to stay in place for 7 days without requiring a change.

In general, total histopathology scores of the treated sites were not substantially higher than those of the + control sites. Epidermal hyperplasia, collagen remodeling, fibroplasia, and vascular proliferation (neovascularization) were noted in all HD-exposed sites (treated and untreated). These are normal processes seen in wound healing,^30^–^34^ and as such it is not surprising that these findings were noted in the tissues collected on PS14 or PS21. Excessive scar tissue formation, which one would only expect to see following deep cutaneous burns that are not excised and grafted, was not observed. In time, these normal physiological responses to tissue injury are expected to subside, resulting in near-normal architecture. Additional studies of a longer duration are needed to judge long-term outcome. While there seemed to be few improvements in histological architecture 14 to 21days following treatment, the reepithelialization process was sped up in many of the Xeroform- and Scarlet Red–treated sites. Also, by the end of the study, skin elasticity of the Xeroform-treated sites was not significantly different from that of sham-exposed skin. If an early dressing change had not been performed in the allograft/Flexzan study (PS04), one may have also seen improved results in that study.

In general, the treated wounds were mildly harder and equally or slightly less elastic than normal skin by 14 to 21days postsurgery. While HD-exposed skin sections were statistically thicker than normal skin, no clinically significant amounts of scar tissue had formed, as seen by histology. In contrast, deep HD injury has been noted to induce relatively thick, hard, and inelastic scar tissue in pigs.^6^,^7^

By the end of each study, the HD-exposed sites (treated and untreated) were mildly erythematous and similar in color to each other. Erythema is a normal short-term consequence of laser resurfacing and subsides with time, especially with Er:YAG lasers.^29^ In all experiments, TEWL rates were highest at PS07 and decreased by PS14. A previous pilot study indicated that barrier function is severely disrupted in ventral pig skin by 3 to 7 days after exposure to liquid HD for 30 or 120 minutes and does not return to baseline values until 63 to 70 days postexposure (J.S.G., unpublished data, 1998). Barrier function principally resides in the stratum corneum. In adult human skin, keratinocytes take approximately 2 to 4 weeks to migrate from the stratum basale to the stratum granulosum and 2 weeks to transit the stratum corneum.^35^ It can thus take 4 to 6 weeks following initial wound coverage (thin confluent layer of keratinocytes) for complete stratification to occur and a disrupted barrier function to return to normal. It is not surprising that barrier function had not yet returned to baseline levels in these pig wounds 14 to 21 days following debridement, especially those sites debrided well into the upper reticular dermis. Nonetheless, signs of barrier repair were noted (eg, decreasing TEWL rates).

Er:YAG lasers have been used for a wide variety of procedures, ranging from facial resurfacing to burn debridement.^29^,^36^–^40^ They have been shown to be particularly useful in the debridement of partial-thickness burns^40^ and in the management of deep Lewisite injuries.^36^ Unlike the Gaussian beam profiles created by CO_2_ lasers, Er:YAG laser beams tend to be uniform and produce uniform depths of ablation.^40^

Biopsies were not collected during surgery to verify that the injuries were partial thickness. An 8-minute exposure to liquid HD was previously shown to consistently produce superficial dermal HD injuries in weanling swine by 48 hours postexposure (F. M. Reid, J. S. Graham, et al, unpublished data, 2004). Histopathological examination of skin samples collected at the end of each study showed the presence of normal hair follicles, glands, and smooth muscles. In addition, hair growth in the lesion areas was noted, and wound contraction after laser debridement absent. These results indicated the HD injuries were not deep-dermal or full-thickness.

These wound healing studies have demonstrated that with appropriate treatment, the healing of cutaneous HD injuries can be improved. Studies in progress are currently evaluating enzymatic and chemical debridement agents as well as a commercially available hydrosurgery system as alternatives to laser debridement. Upcoming wound healing experiments will examine the effect of less aggressive laser debridement (eg, to a depth of 100 μm), and the efficacy of skin substitutes, matrix metalloprotease-binding dressings, silver-ion delivering dressings, topical nutritional support, keratinocyte sprays, and vacuum-assisted closure. Screening a variety of debridement protocols and treatment adjuncts is crucial in determining the most efficacious treatment regimen to be applied in the clinical management of HD casualties. Improved treatment will speed the rate of wound closure, result in a better cosmetic and functional outcome for the patient, and will enable the casualty to return to normal activities sooner.

## CONCLUSIONS

The conclusions of the study can be listed as follows:

- Within the first week of surgery, the neoepidermis appears to be very fragile and easily removed. Care must be taken during bandage changes, and a nonadherent dressing that could be left in place for a longer period of time (eg, 7 days) would be beneficial, both to the patient and medical logistical burden (eg, nursing care).
- The use of cultured epithelial allograft material may have a potential role in the treatment of debrided cutaneous HD injuries if grown on a completely nonadherent backing and left undisturbed for at least a week.
- Following laser debridement, Xeroform Petrolatum and Scarlet Red Ointment dressings are effective and inexpensive dressings for treatment of cutaneous HD injury. Reepithelialization was nearly complete by 7 days postsurgery for many of the debrided sites treated with these dressings.

## ACKNOWLEDGMENTS

The authors thank MAJ Stephen Dalal, DVM, SPC Shane Warren, and Tabatha Venker for veterinary support; F. Steven Tucker and staff for clinical pathology support; Jamie Martin and staff for histology support; and Bruce Freedman, MD, FACS (Plastic Surgery Associates of Northern Virginia, LTD, McLean, Va), for guidance in use of the Sciton Profile laser.
